# Extracellular DNA as a genetic recorder of microbial diversity in benthic deep-sea ecosystems

**DOI:** 10.1038/s41598-018-20302-7

**Published:** 2018-01-30

**Authors:** C. Corinaldesi, M. Tangherlini, E. Manea, A. Dell’Anno

**Affiliations:** 1 Dipartimento di Scienze e Ingegneria della Materia, dell’Ambiente ed Urbanistica, Polytechnic University of Marche, Via Brecce Bianche, 60131 Ancona, Italy; 2 Dipartimento di Scienze della Vita e dell’Ambiente, Polytechnic University of Marche, Via Brecce Bianche, 60131 Ancona, Italy; 3 Stazione Zoologica A. Dohrn, Villa Comunale, Naples, Italy

## Abstract

Extracellular DNA in deep-sea sediments represents a major repository of genes, which previously belonged to living organisms. However, the extent to which these extracellular genes influence current estimates of prokaryotic biodiversity is unknown. We investigated the abundance and diversity of 16S rDNA sequences contained within extracellular DNA from continental margins of different biogeographic regions. We also compared the taxonomic composition of microbial assemblages through the analysis of extracellular DNA and DNA associated with living cells. 16S rDNA contained in the extracellular DNA pool contributed up to 50% of the total 16S rDNA copy number determined in the sediments. Ca. 4% of extracellular Operational Taxonomic Units (OTUs) were shared among the different biogeographic regions revealing the presence of a core of preserved OTUs. A higher fraction of OTUs was exclusive of each region potentially due to its geographic and thermohaline characteristics. Ca. one third of the OTUs identified in the extracellular DNA were absent from living prokaryotic assemblages, possibly representing the signatures of past assemblages. Our findings expand the knowledge of the contribution of extracellular microbial sequences to current estimates of prokaryotic diversity obtained through the analyses of “environmental DNA”, and open new perspectives for understanding microbial successions in benthic ecosystems.

## Introduction

Extracellular DNA in surface deep-sea sediments is by far the largest reservoir of DNA of the world oceans^1^. The main sources of extracellular DNA in such ecosystems are represented by *in situ* DNA release from dead benthic organisms, and/or other processes including cell lysis due to viral infection, cellular exudation and excretion from viable cells, virus decomposition, and allochtonous inputs from the water column^1–4^. Previous studies provided evidence that an important fraction of extracellular DNA can escape degradation processes, remaining preserved in the sediments^5,6^. This DNA represents, potentially, a genetic repository that records biological processes occurring over time^7,8^.

Recent investigations revealed that DNA preserved in marine sediments is characterized by a large number of highly diverse gene sequences^7–10^. In particular, extracellular DNA has been used to reconstruct past prokaryotic and eukaryotic diversity in benthic ecosystems characterized by low temperatures and/or permanently anoxic conditions^10–14^.

Previous studies suggested that the preservation of DNA might be also favoured in benthic systems characterised by high organic matter inputs and sedimentation rates, such as continental margins^15,16^. These systems, which represent ca. 15% of the global seafloor, are also hotspots of benthic prokaryotic diversity^17–19^, and therefore they could represent optimal sites to investigate the prokaryotic diversity preserved within extracellular DNA.

Spatial distribution of prokaryotic diversity has been intensively studied in benthic deep-sea ecosystems^20–23^ through the analysis of “environmental DNA” (i.e., the genetic material obtained directly from environmental samples without any obvious signs of biological source material^24^). However, the extent to which gene sequences contained within extracellular DNA can alter the estimates of the diversity of the present-day prokaryotic assemblages is unknown^25^.

In the present study, we utilised the extracellular DNA pool as a recorder of the prokaryotic diversity in the sediments of different sites of continental margins (Atlantic and Arctic Ocean, and Mediterranean Sea). The prokaryotic genetic signatures contained in the different extracellular DNA pools were compared among them, and with the gene sequences belonging to living microbial assemblages. Findings reported here provide new insights on ubiquitous and exclusive prokaryotic signatures preserved in different biogeographic regions, and their contribution to the estimates of the current diversity.

## Results

### Environmental variables

Temperature ranged from −0.84 °C to 13.1 °C (in the Arctic and Mediterranean sites, respectively) and salinity from 34.84 to 38.49 (in the NE Atlantic 1 and Mediterranean sites, respectively). The lowest concentrations of biopolymeric carbon were found in the site NE Atlantic 2 whereas the highest one in the Arctic margin (1.33 ± 0.17 and 4.14 ± 0.53 and mgC g^−1^ of sediments, respectively, Table S1).

### Total extracellular DNA concentrations and 16S rDNA copy number

**Figure 1:**
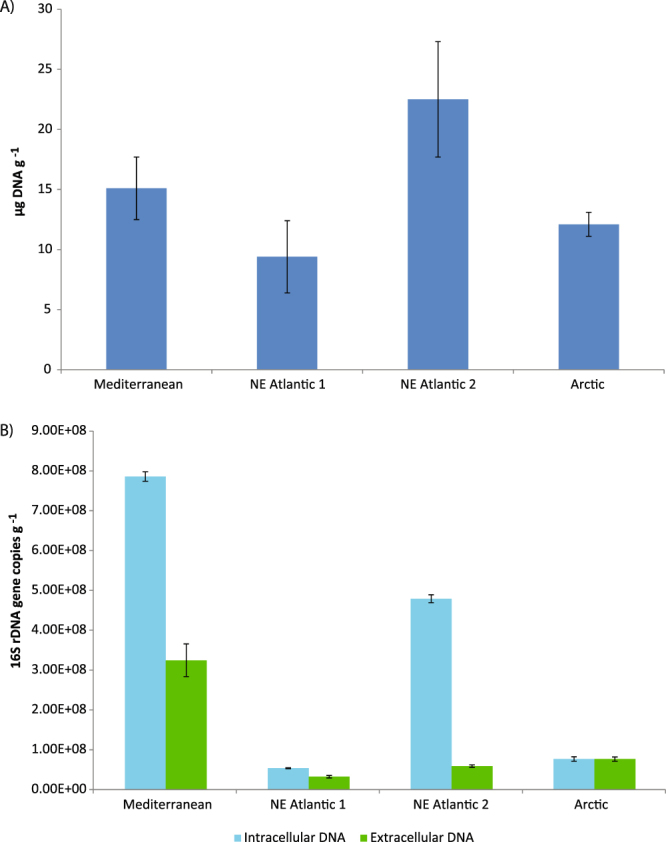
Total extracellular DNA concentrations in the sediments of continental margins from NE Atlantic and Arctic Oceans and Mediterranean Sea (**A**); copy number of 16S rRNA genes in the extracellular and intracellular DNA pools from the sediments of continental margins (**B**).

The concentrations of total extracellular DNA in surface sediments of the continental margins investigated ranged from 9.4 ± 3.0 μg DNA g^−1^ to 22.5 ± 4.8 μg DNA g^−1^ (in the sites NE Atlantic 1 and 2, respectively; Fig. 1A). The concentrations of total extracellular DNA determined in the deepest site of the NE Atlantic 2 were significantly higher than those of NE Atlantic 1 and Arctic sites (p < 0.01), but not significantly different from those of the Mediterranean Sea.

The copy number of 16S rDNA ranged from 3.2 ± 0.3 × 10^7^ to 32.5 ± 4.1 × 10^7^ g^−1^ within the extracellular DNA pools (in the NE Atlantic 1 and Mediterranean sites, respectively) and from 5.4 ± 0.2 × 10^7^ to 78.5 ± 1.2 × 10^7^ g^−1^ within the intracellular DNA pools (in the NE Atlantic 1 and Mediterranean Sea, respectively; Fig. 1B). The copy number of 16S rDNA contained in the extracellular and intracellular DNA pools varied significantly among all the samples investigated (p < 0.01). In all benthic sites the abundances of prokaryotic 16S rDNA in the extracellular DNA pools were significantly lower than in the intracellular DNA pools (p < 0.01), except for the Arctic site where no significant differences were found.

The contribution of extracellular 16S rRNA gene copies to the total pool of 16S rDNA copies (as the sum of 16S rRNA gene copies contained in extracellular and intracellular DNA pools) ranged from 11% to 50%, in NE Atlantic site 2 and Arctic site, respectively.

### Prokaryotic diversity associated with extracellular DNA pools

The number of sequences obtained for each extracellular DNA sample at each step of quality check and during the QIIME analysis are reported in Table S2.

The number of OTUs after normalisation to the same number of sequences ranged from 1003 to 1158 in the NE Atlantic 2 and Mediterranean samples, respectively (Table S3, Figure S1).

Extracellular DNA pools from all sites investigated were characterized by 16S rDNA gene sequences affiliating to a number of prokaryotic families ranging from 201 (in the Mediterranean Sea) to 236 (NE Atlantic 1; Table S3). Overall, 316 families were identified in the extracellular DNA pools, 39% of which were shared among all pools. From ca. 4% to 8% of the families were exclusive of each site (in Arctic and NE Atlantic 2 sites, respectively).

**Figure 2:**
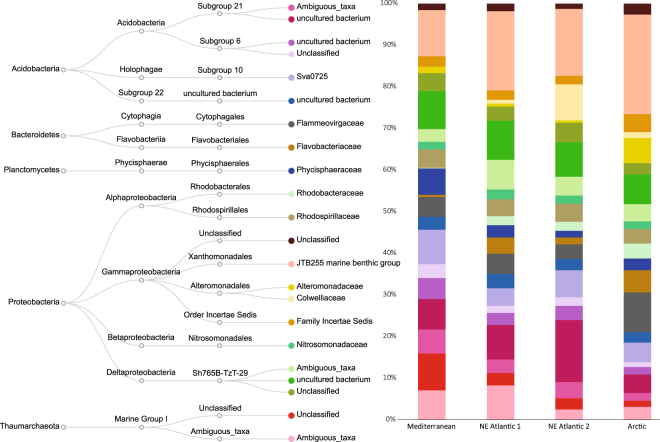
Taxonomic composition of prokaryotic assemblages in the extracellular DNA pools from deep-sea benthic ecosystems of continental margins. The taxa, whose OTUs contribute for at least 1% to the whole prokaryotic assemblages, are shown.

Prokaryotic assemblages were mainly represented by JTB255 marine benthic group (Gammaproteobacteria), which contributed from 6% to 14% to the total number of sequences, unclassified Sh765B-TzT-29 (Deltaproteobacteria, from 8% to 12%) and uncultured bacterium of Subgroup 21 (Acidobacteria, from 4% to 9%), followed by other bacterial and archaeal families including *Sva0725* (Subgroup 10, Acidobacteria), *Flammeovirgaceae* (Cytophagales, Bacteroidetes) and unclassified/ambiguous taxa belonging to Marine Group I (Thaumarchaeota, Fig. 2).

**Figure 3:**
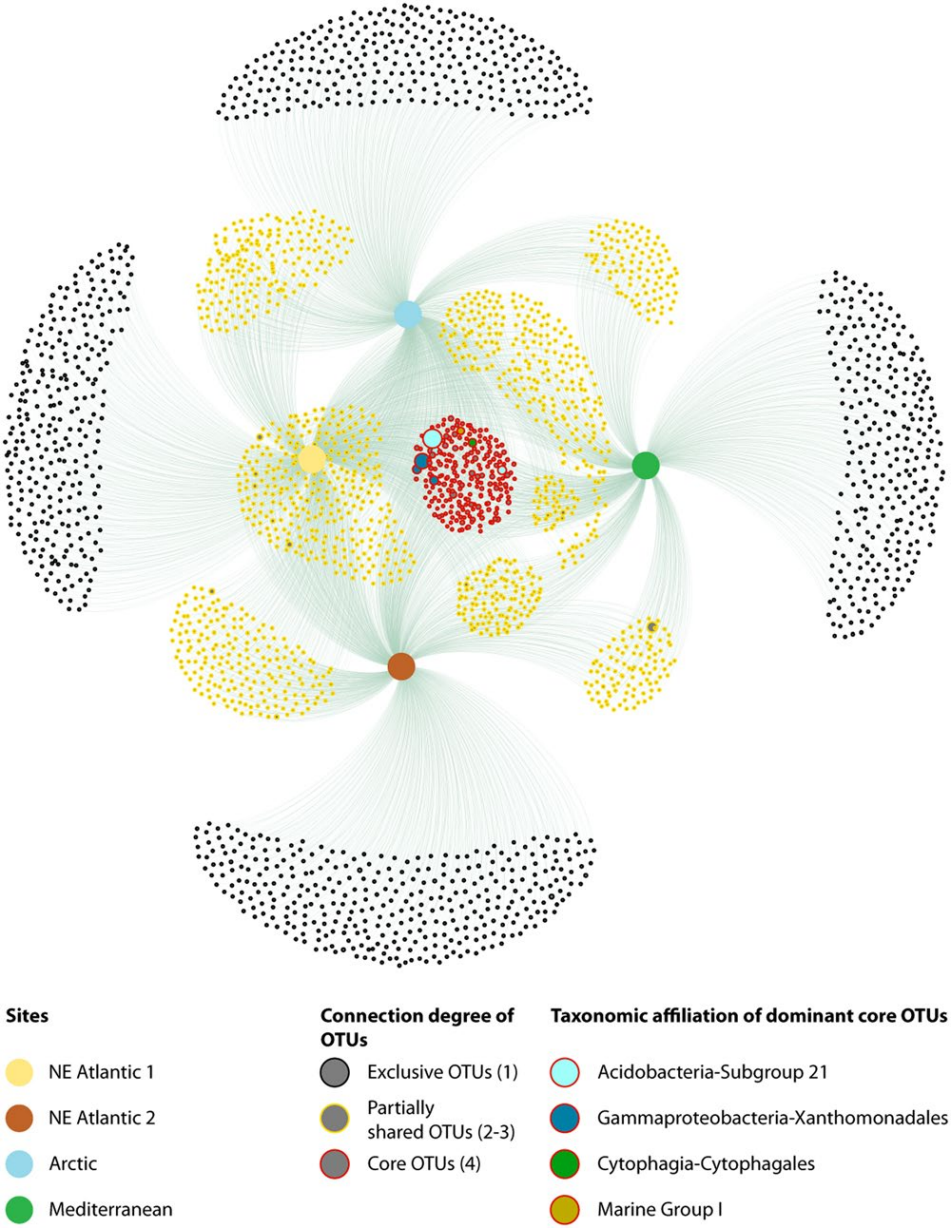
OTU network of the extracellular DNA pools from the sediments of the different continental margins. Dot size within the network is proportional to the abundance of sequences for each OTU. Dots circled in red represent extracellular core OTUs, dot circled in yellow are partially shared (among two or more pools) OTUs, dots circled in black are OTUs exclusive of each pool. The core OTUs contributing at least for 20 sequences are shown. The numbers in parentheses represent the number of connections among OTUs and samples: 1 for exclusive OTUs, 2–3 for partially shared OTUs and 4 for core OTUs.

The network-based analysis showed the presence of core OTUs (i.e. shared among all the different extracellular DNA pools) belonging to different taxa mainly affiliated with Acidobacteria Subgroup 21, *Xantomonadaceae* and JTB255 marine benthic group (Fig. 3). Such core OTUs accounted for only ca. 4% to the total OTU number. This analysis also showed the presence of a large fraction of partially shared OTUs (i.e., shared among two or three DNA pools) contributing for ca. 61% to the total number of OTUs. Finally, all extracellular DNA pools were characterized by several OTUs exclusive of each site, accounting, on average, for 35% to the total number of OTUs.

SIMPER analyses revealed that prokaryotic assemblages contained within the extracellular DNA pool from the Mediterranean sample were largely dissimilar from those from NE Atlantic 1 or Arctic samples (ca. 58–66%).

The results of the cluster analysis based on the Jaccard dissimilarity indicated that the different extracellular DNA pools (in terms of OTU presence/absence) from NE Atlantic 1 and NE Atlantic 2 sites clustered together whereas Arctic and, particularly, the Mediterranean samples were separated (Figure S2).

### Comparison between the prokaryotic diversity associated with extracellular and intracellular DNA pools

The number of sequences obtained for each extracellular and intracellular DNA sample at each step of quality check and during the QIIME analysis are reported in Table S2. The OTU richness and number of families (obtained using the same number of sequences) identified within each DNA pool are reported in Table S4 while rarefaction curves are shown in Figure S1.

Intracellular DNA pools from the NE Atlantic sites were characterized by 16S rDNA gene sequences belonging to a total of 262 prokaryotic families in both sites (Figure S3) while prokaryotic signatures contained in the extracellular DNA pools were affiliated with 268 families. Xanthomonadales, Deltaproteobacteria and Acidobacteria were the dominant prokaryotic taxa either in intracellular or extracellular DNA pools.

**Figure 4:**
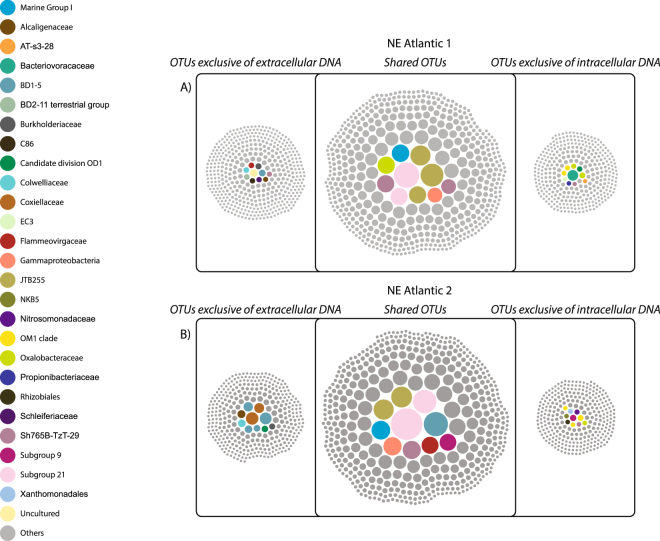
OTUs shared between extracellular and intracellular DNA pools and exclusive OTUs of each pool from the sediments of NE Atlantic 1 (**A**) and NE Atlantic 2 (**B**) sites. Dot size is proportional to the contribution of sequences to each OTU belonging to the different prokaryotic families. The OTUs, which contribute most to the prokaryotic assemblage in terms of sequences, are colored according to their taxonomic affiliation (at the lowest identifiable taxonomic level).

In the NE Atlantic sites 1 and 2, 43% and 46% of the prokaryotic OTUs (mainly affiliated with Subgroup 21, Sh765B-TzT-29 and JTB255) were respectively shared between extracellular and intracellular DNA pools (Fig. 4A,B). Ca. one third of the prokaryotic OTUs were exclusively present in extracellular DNA from each of the two NE Atlantic sites.

In the NE Atlantic site 1, we found some exclusive prokaryotic taxa of extracellular DNA pool belonging to *Burkholderia* and Thermoplasmatales, whereas no exclusive family was found in the intracellular DNA pools (Fig. 4A).

In the NE Atlantic site 2, exclusive prokaryotic taxa of the intracellular DNA pool mainly belonged to TM6 (although their sequence contribution to the whole assemblage was <0.1%), whereas taxa affiliated to *Burkholderiaceae* and OD1 mainly belonged to the extracellular DNA pool (Fig. 4B).

Cluster analyses indicated that the assemblages of prokaryotic OTUs of the extracellular and intracellular DNA pools in both the NE Atlantic sites clustered together although with a high level of dissimilarity (ca. 52% on average, as calculated by the SIMPER analysis and 64.5% with binary Jaccard dissimilarity).

The analysis of intracellular DNA pools showed that a set of OTUs were shared from the two pools from the NE Atlantic sites (6.5%). This fraction was very similar to the percentage of core OTUs of the extracellular DNA pools from the two NE Atlantic sites (7.4%). Core OTUs of the intracellular DNA pools belonged to the same prokaryotic taxa as the core OTUs of extracellular DNA pools (JTB255, Acidobacteria Subgroup 21, Xantomonadaceae).

## Discussion

Previous studies suggested that the preservation of DNA in deep-sea sediments may be favoured by an array of factors including anoxic conditions, low temperatures, and high sedimentation rates, as observed in continental margins^6,15^. In the present study, we found that the total extracellular DNA concentrations, in the sediments of different continental margins, were among the highest values reported so far for deep-sea ecosystems^1,2^. In particular, we provide evidence that the deepest sediments among those investigated (4902 m depth in the Atlantic Ocean) were characterized by the highest amount of extracellular DNA, suggesting that benthic ecosystems along continental margins, even at abyssal depth, can be a major repository of extracellular DNA.

Investigations conducted in subsurface anoxic sediments and in surface sediments of coastal and deep-sea ecosystems revealed that extracellular DNA can preserve sequences of dead prokaryotes and eukaryotes^7,8,25^. Our findings obtained from surface sediments of continental margins show that extracellular 16S rRNA gene copies accounted for a variable fraction of the total number of 16S rRNA gene copies (as the sum of extracellular and intracellular 16S rDNA gene copies) depending on the investigated site. The number of 16S rRNA gene copies contained in the extracellular DNA pool were not correlated to the total extracellular DNA concentrations. This could be explained by the heterogeneous composition of the extracellular DNA pool, which includes genes different from the 16S rDNA, and/or its level of fragmentation/damage, which is influenced by a variety of biotic and abiotic factors^25^.

We also found that the extracellular 16S rDNA sequences were highly diverse and affiliated to both archaeal and bacterial families, most of which have not been classified yet.

More than one third of the prokaryotic families were common to all of the extracellular DNA pools. These families, including those affiliated with the unclassified Subgroup 21 Acidobacteria, the unclassified Sh765BTz-29 Deltaproteobacteria and JTB255 marine benthic group (Xanthomonadales), are typical of benthic deep-sea ecosystems^26–29^. However, the analysis conducted at the OTU level revealed that the similarity among different extracellular DNA pools was very low, suggesting the presence of a low but highly represented (in terms of sequence abundance) number of core OTUs^28–30^. This was also highlighted by the network analysis, which showed a core of extracellular OTUs (mainly affiliated with Acidobacteria Subgroup 21 and JTB255 marine benthic group), characterized by the highest connectivity among samples. Such OTUs were thus present in all biogeographic regions despite their different temperature and salinity values, trophic conditions, sedimentation rates and consequently age of the sediment layer considered (from decades to several hundreds of years). These findings allow us to hypothesize that the prokaryotic core signatures might belong to resident taxa in all sites of continental margins for several decades. On the other hand, we also observed the presence of groups of extracellular OTUs exclusive of each biogeographic region suggesting that different environmental conditions could select specific taxa^31^ and/or favour the preservation of their genetic signatures^12,25^. We found that extracellular OTU pools from the two sites of the NE Atlantic margin grouped together, whereas the extracellular OTU pool from the Mediterranean site clustered apart, potentially due to the wide differences in temperature and salinity regimes. Since the sedimentation rates determined in the two NE Atlantic sites were very different, we exclude that the age of the sediment, in the order of hundreds of years, can represent a key factor influencing the preservation of extracellular prokaryotic signatures as previously reported in other benthic ecosystems^25^.

The comparison between extracellular and intracellular DNA pools revealed that they contained a rather similar number of OTUs, which fall within the range of values reported so far for benthic deep-sea ecosystems^27,32^. Ca. one third of the total OTUs were shared between extracellular and intracellular DNA pools in each site indicating a low level of similarity of the composition of prokaryotic assemblages contained therein (as also revealed by the Jaccard index). Indeed, we observed a high fraction of OTUs exclusive either of the intracellular DNA or of the extracellular DNA, which even contained OTUs affiliated to families not encountered in any of the two intracellular DNA pools of the Atlantic margin (such as those belonging to TM6, Burkholderiales and others).

The low level of similarity between OTUs of the extracellular and intracellular DNA pools cannot be attributed to methodological artefacts/biases related with cell lysis induced by the procedure here used, and/or sequencing effort applied to our samples. Indeed, the analyses performed indicate that the amount of intracellular DNA/prokaryotic genes released due to sample manipulation and/or active growth of the cells is negligible^33^. At the same time, despite the sequencing effort in our samples was not completely exhaustive, the rarefaction curves obtained were not far from reaching the saturation, suggesting that prokaryotic diversity was largely described. Therefore, the presence of OTUs exclusive of the intracellular DNA pool could be explained by different degradation rates of the prokaryotic sequences once outside the cells or the sporadic inputs of microbial taxa from the water column^8,32^. On the other hand, the presence of prokaryotic taxa exclusively found in the extracellular DNA pool suggests that they are no longer part of the living microbial fraction possibly representing the signatures of past assemblages^8,12^.

The exclusive extracellular prokaryotic taxa found in our study have been already reported in other benthic ecosystems through extraction procedures of total sedimentary DNA (without discriminating between DNA associated with extracellular and microbial fractions^34,35^). However, in these cases, it cannot be excluded that, due to the potential co-extraction of past prokaryotic signatures, the current diversity has been over-estimated. Therefore, we claim for the need of considering the contribution of extracellular DNA in the current estimates of benthic prokaryotic biodiversity obtained through the analyses conducted on the so called “environmental DNA“^24^. Moreover, the contextual analysis of prokaryotic signatures associated with intracellular DNA and extracellular DNA might represent a useful and effective tool for understanding microbial successions from the past to present-day.

## Materials and Methods

### Study areas and sampling strategy

Surface sediment samples were collected using a multiple corer in different continental margins of the Mediterranean Sea, NE Atlantic and Arctic Oceans. In particular, sediment samples were collected in the NW Mediterranean Sea at 2342 m depth (42.080 N, 4.682E), in the NE Atlantic Ocean, at two sites at 3475 m (40.167 N, 9.983E, hereafter defined NE Atlantic 1) and 4902 m depth (40.167 N, 10.984E; hereafter defined NE Atlantic 2) and in the Arctic Ocean at 2482 m depth (79.067 N, 4.170E; Table S1).

At each site, three sediment cores were collected by independent deployments (n = 3) of multiple corers, which allow the collection of hermetically-closed samples. Immediately after retrieval, the sediment cores were sliced vertically and the top layer (0–2 cm) was stored at −80 °C until laboratory analyses. Moreover, at each site temperature and salinity of bottom waters were measured by CTD.

The high variability in the sedimentation rates across the investigated sites (Table S1) resulted in different ages (i.e., 12 years for the Mediterranean site, 67 and 667 years for the sites NE Atlantic 1 and 2, respectively, and 105 years for the Arctic site) of the sediments considered^25^.

### Biochemical composition of organic matter

The concentrations of proteins, carbohydrates and lipids in the sediment were determined spectrophotometrically^36^ and expressed as bovine serum albumin, glucose and tripalmitine equivalents, respectively. The sum of the carbohydrate, protein and lipid concentrations converted into carbon equivalents (using the conversion factors of 0.40, 0.49 and 0.75 mg C mg^−1^, respectively) was defined as the biopolymeric organic carbon^37^.

### Total extracellular DNA concentrations

Working conditions and precautions during extracellular DNA analyses are described in Supplementary Information.

To determine the concentrations of total extracellular DNA in the sediment we used a procedure based on the hydrolysis of the extracellular DNA (using commercial nucleases) which does not allow the recovery of the DNA for subsequent molecular studies^25,38,39^; therefore, contextual aliquots of the same samples were also processed to provide extracellular DNA that was suitable for molecular analyses.

For the total extracellular DNA analyses, 2.5 mL 0.1 M Tris, 0.1 M NaCl, 1 mM CaCl2, 10 mM MgCl2, pH 7.5, was added to 1 g sediment (wet weight; i.e., buffer: sediment ratio of 2.5:1 [v/w]). Aliquots of the sediment samples (n = 3) had additions of DNase I (1.9 U mL^−1^), nucleases P1 and S1 (4.0 and 2.3 U mL^−1^, respectively), and exonuclease-3 (1.9 U mL^−1^); another set of replicates was added to an equal volume of buffer (without the enzymes) and used as a control. The samples were incubated at room temperature for 2 h under gentle agitation, and then centrifuged at 2,000 × g for 5 min, with the resultant supernatants used to determine the DNA released from the sediments. The supernatants were dried under vacuum and analysed fluorometrically using diaminobenzoic acid. The fluorescence of the hydrolysed DNA was converted into concentrations using calibration curves obtained from standard solutions of *Escherichia coli* DNA (from 0.05 to 5.0 µg DNA mL^−1^). The amounts of extracellular DNA hydrolysed by the nucleases were obtained from the differences between the DNA concentrations of the enzyme-treated samples and the control samples.

The extracellular DNA concentrations were expressed as micrograms of total extracellular DNA per gram dry sediment.

### Extraction of extracellular and intracellular DNA pools suitable for molecular analyses

The extracellular and intracellular DNA used for molecular analyses were recovered contextually from the same sediment sample following the protocol developed by Corinaldesi *et al*.^39^, with some modifications to increase the extraction efficiency. The robustness and reliability of this protocol for extracellular DNA extraction has been shown to exclude any possible contamination due to cell lysis for co-extraction of DNA contained in the microbial cells^10,39^. Briefly, the sediment samples were treated with acid-washed polyvinylpolypyrrolidone (0.05% final concentration) and SDS (final concentration, 0.1%). Then the samples were chilled on ice, centrifuged, and the supernatants were transferred to sterile tubes. The sediment pellets were washed again with sodium phosphate buffer (pH 8.0) and centrifuged. These steps were repeated 6 times. Supernatants were combined and centrifuged for 20 min at 10000 × g (4 °C). After centrifugation, the supernatants containing extracellular DNA were filtered through 0.02-µm-pore-size filters to eliminate any contaminating viruses or prokaryotic cells. Aliquots of the supernatant fluid after filtration were further checked using epifluorescence microscopy to exclude viral or prokaryotic contamination. The pellets containing prokaryotic cells were treated with DNase I to exclude any possible contamination from residual extracellular DNA, and then processed for DNA extraction by using the UltraClean soil DNA isolation kit (MoBio Laboratories Inc., CA, USA) according to the manufacturer’s instructions.

The extracellular DNA contained in the supernatant fluid was recovered by adding 1 volume of cetyltrimethylammonium bromide solution (1% CTAB in 50 mM, Tris 10 mM EDTA, pH 8.0) and further precipitation after incubation at 65 °C for 30 min, cooling on ice and centrifugation at 10000 × g for 15 min at 4 °C. The pellet was suspended in high-salt TE buffer (pH 8.0) added to 0.6 volumes of cold isopropanol, incubated for 2 h at −20 °C and centrifuged at 10000 × g for 15 min at 4 °C. The pellets containing the extracellular DNA were suspended in MilliQ water and purified using the Wizard PCR clean-up system (Promega).

The details of the analyses conducted to test the absence of cell lysis potentially induced by the procedure described above are provided in the Supplementary Information.

### Quantitative PCR analyses

Quantitative PCR (qPCR) analyses of the prokaryotic 16S rDNA gene copies were carried out both in the extracellular and intracellular DNA pools that were extracted contextually from the same sediment samples^39^. qPCR analyses were performed using the TaqMan technology^40^. The prokaryotic 16S rDNA sequences were amplified using the universal primers Uni340F (5′-CCTACGGGRBGCASCAG-3′) and Uni806R (5′-GGACTACNNGGGTATCTAAT-3′). The TaqMan probe was Uni516F (5′-TGYCAGCMGCCGCGGTAAHACVNRS-3′), which contained a fluorescent reporter dye (6-carboxyfluorescein) covalently attached to the 5′-end, and a fluorescent quencher dye (6-carboxytetramethylrhodamine) attached six or more bases downstream of the reporter dye^41^. All of the real-time PCR was performed in a volume of 25 μL with an iQ5-icycler (Bio-Rad) using iQ Supermix (2×; Bio-Rad) containing 40 mM Tris-HCl, pH 8.4, 100 mM KCl, 0.4 mM each dNTP (dATP, dCTP, dGTP, dTTP), 50 U/mL hot-start iTaq DNA polymerase, and 6 mM MgCl_2_. To amplify prokaryotic genes, 40 PCR cycles were used: as 95 °C for 15S, and 57 °C for 5 min, which were preceded by 3 min of Taq activation at 95 °C. Negative controls were performed by using the same reaction mixture without DNA template. To quantify the 16S rDNA, calibration curves were obtained from a standard solution of *E. coli* (from 0.2 to 200 pg µL^−1^). Standard concentrations were plotted against the number of cycles at which the fluorescence signal increased above background, or the cycle threshold (the Ct value). The iCycler software analysis programme was used to calculate the Ct values and to determine the sample concentrations based on the standard curves. The copy number of ribosomal gene sequences determined by qPCR was normalised to sediment dry weight for a comparison with available literature information^42^.

### Sequencing and bioinformatics

Genetic diversity of the prokaryotic 16S rDNA sequences associated with the extracellular DNA pools was analysed in the sediment of all areas. In addition, we analysed 16S rDNA diversity within the intracellular DNA pool in the two sites of NE Atlantic Ocean. Analyses were conducted by tag-encoded amplicon pyrosequencing of hypervariable regions (from V5 to V9). Bacterial and archaeal 16S rDNA amplicons were generated using the universal primers 789 F (5′ -TAGATACCCSSGTAGTCC-3′) and 1492 R (5′ –GGTTACCTTGTTACGACTT-3′)^43^ and sequenced on a Genome Sequencer 454 FLX Titanium platform (Roche). Three independent PCR analyses were carried out from each replicated extraction (n = 3) of extracellular and intracellular DNA. The amplicons obtained from PCR analysis of extracellular DNA were pooled together as well as those for intracellular DNA. Additional details on pyrosequencing analyses are reported in Supplementary Information.

Raw sequences were first subjected to homopolymer removal by means of the Acacia tool^44^ with standard values, and subsequently quality-trimmed by means of the PRINSEQ tool^45^ by removing sequences with a mean quality score <20 and further filtered to remove sequences shorter than 100 bp. The number of sequences obtained through each step is reported in Table S2. High-quality amplicon reads were subsequently analysed by the QIIME pipeline^46^, aligning them against the SILVA database (v119)^47^ by means of the PyNAST aligner using the open-reference strategy^48^ and clustering them at 97% of identity. The ChimeraSlayer tool was used to assess the presence of chimeric sequences, which were not found in any sample.

All bioinformatic analyses were performed using the same number of sequences to properly compare the different samples. In particular, we used 3400 sequences for comparing extracellular DNA pools from different geographical areas (Mediterranean, Arctic and NE Atlantic margins) and 5000 sequences for comparing intracellular and extracellular DNA pools obtained contextually from the two sites of the NE Atlantic margin (the NE Atlantic 1 and 2 sites).

OTU networks were created on the data provided by the *make_otu_network* script provided by the QIIME pipeline with standard values on the extracellular DNA samples with the *gephi* tool^49^ by means of a combination of the Yifan-Hu and Force Atlas 2 algorithms provided by the program, with the Dissuade Hubs and Prevent Overlap flags toggled for ease of visualization.

Comparative analyses between assemblage composition of extracellular and intracellular DNA pools from the sediments of NE Atlantic 1 and 2 sites were carried out to evaluate the contribution of preserved OTUs to the total prokaryotic diversity. To do so we determined exclusive taxa contained within extracellular and intracellular DNA pools of the same site by counting OTUs exclusively found in either extracellular or intracellular DNA pools.

### Statistical analyses

Analyses of variance (ANOVA) were carried out to test for differences in the extracellular DNA concentrations and prokaryotic 16S rDNA copy number among sampling sites. SIMPER analyses were carried out to assess the similarity among the prokaryotic OTUs contained within the different extracellular DNA pools and between extracellular DNA and their respective intracellular DNA pools^50^. All analyses were carried out with the PRIMER-E 6 suite^50^. In addition, to assess dissimilarity among extracellular DNA pools and between extracellular DNA and their respective intracellular pools (in terms of presence/absence of OTU), the binary Jaccard distance was determined by means of the *beta_diversity* script within QIIME and the results visualized with an UPGMA tree^46^.

### Data accessibility

The sequences have been submitted to the MG-RAST server under the project “Continental Margins” accessible for reviewers at the following token: http://metagenomics.anl.gov/mgmain.html?mgpage=token&token=67EuldY6_FfX8tRaYFO8Qxlo6LPnMq8OO2phSra8KwIvMUjy0m.

## Electronic supplementary material

Supplementary Information
