# Supplementary material for: Extracellular DNA as a genetic recorder of microbial diversity in benthic deep-sea ecosystems
Source: Sci Rep. 2018 Jan 30;8:1839. doi: 10.1038/s41598-018-20302-7 (PMC5789842; doi:10.1038/s41598-018-20302-7)
Supplement: Supplementary file 1 — Supplementary Information [file 41598_2018_20302_MOESM1_ESM.doc]

**AMENDED VERSION**

**(SREP-17-29215)**

**Supplementary information**

**Extracellular DNA as a genetic recorder of microbial diversity in benthic deep-sea ecosystems**

Corinaldesi C.1­, Tangherlini M.2,3, Manea E.2, Dell’Anno A.2

1Dipartimento di Scienze e Ingegneria della Materia, dell’Ambiente ed Urbanistica, Polytechnic University of Marche, Via Brecce Bianche, 60131 Ancona, Italy

2Dipartimento di Scienze della Vita e dell’Ambiente, Polytechnic University of Marche, Via Brecce Bianche, 60131 Ancona, Italy

3Stazione Zoologica A. Dohrn, Villa Comunale, Naples, Italy

**Supplementary materials and methods**

**Supplementary tables**

**Supplementary figures**

**Supplementary references**

Supplementary materials and methods

*Working conditions and precautions during extracellular DNA analyses*

To avoid contamination of extracellular DNA by exogenous nucleic acids during sediment sampling and handling, stringent precautionary conditions were adopted. The sediment core was sliced by using sterile tools and gloves, and facemasks. The analyses of extracellular DNA (extraction, quantification and PCR) were conducted in a clean laboratory, which was exclusively dedicated to this purpose. All the procedures were conducted under a laminar-flow hood, and the surfaces and instruments used were frequently cleaned with bleach. All of the glassware used was carefully cleaned by soaking it in 1 N NaOH, 10% HCl, and then in MilliQ water, with subsequent treatment at 250 °C. The solutions used were prepared with prefiltered MilliQ water, and then were autoclaved and filtered through 0.02 μm pore-size filters (Anodisc, Whatman) to remove potential viral and cellular contaminations.

The PCR analyses were performed under a sterile hood, using sterile solutions, materials, tools and instruments. The operators during the analyses wore whole body protection, gloves and a facemask.

Negative controls were used to monitor possible contamination at each analytical step. To assess possible contamination of extracellular DNA with foreign DNA in the laboratory, additional sediment sub-samples (five replicates for each extraction) previously pre-combusted in a muffle furnace (450°C for 2 h), and then autoclaved were used as negative controls. For PCR analyses, negative controls, which contained the PCR mixture without the DNA template, were run (performed in a ratio of 1:1).

*Testing for the absence of cell lysis*

The robustness and reliability of our protocol for extracellular DNA extraction was tested through several experiments to exclude any contamination due to cell lysis. To do so, pure cultures of the marine bacterium *Vibrio mediterranei* were mixed with sediment sub-samples that had been pretreated in the muffle furnace at 450 °C for 2 h. *V. mediterranei* was grown in marine broth (Difco) and collected during the exponential growth phase (about 5 × 10^8^ cells mL-1, as measured by optical density at 600 nm). The bacterial suspension was pretreated with 10 U DNase I mL-1 (Sigma) for 15 min at 37 °C to avoid the potential release of extracellular DNA during the exponential growth of *V. mediterranei*. After incubation, the bacterial suspension was filtered through 0.2-μm-pore-size Nucleopore filters, and washed three times with 5 ml artificial seawater, to remove the DNase. The filters retaining *V. mediterranei*, which were mixed with sediment sub-samples (two replicates) pre-treated in the muffle furnace (450 °C for 2 h), were processed for the recovery of extracellular DNA. The pellet (containing the bacterial cells) was separated from the supernatant, and used for DNA extraction from intact cells. To determine whether the handling and chemical treatments caused prokaryotic cell lysis, we checked for extracellular DNA in the supernatant. To do this, an aliquot of the supernatant was precipitated with CTAB buffer (1% CTAB in 50 mM Tris, 10 mM EDTA, pH 8.0) and subsequently processed by using the procedure described in the main text of the manuscript. Moreover, the reliability of the procedure for extracellular DNA recovery was tested by adding 1 μg of purified DNA from *V. mediterranei* to another aliquot of the supernatant before CTAB precipitation. The presence of the 16S rDNA sequences in the extracellular and intracellular DNA fraction was investigated by qPCR by using the TaqMan technology targeting the 16S rDNA sequences as described in the main text of the manuscript. These experiments confirm the lack of extracellular DNA release from cell lysis since no contamination was detected in the supernatants, considering a detection limit of the qPCR experimentally determined of 25-30 ribosomal gene copies.

In addition, we carried out another set of experiments to exclude the possible contamination of the extracellular DNA pool with the intracellular DNA due to cell lysis induced by sample freezing at -80°C (to enable the sample preservation) and the subsequent thawing (to enable the sample analyses). We tested the potential release of extracellular DNA from pure cultures of the marine bacterium *Vibrio harveyi,* which was grown in marine broth (Difco), and collected in the exponential growth phase (about 1 × 10^8^ cells ml-1, as measured by optical density at 600 nm). The bacterial suspension was pretreated with 10 U of DNase I (Sigma) ml-1 for 15 min at 37°C, filtered through 0.2-µm-pore-size Nuclepore filters and the filters were mixed with sediment sub-samples (three replicates) pre-treated in the muffle furnace. Samples were then frozen at -80°C for 1 week and subsequently thawed for the analysis of the extracellular DNA according to the protocol described in the main text of the manuscript. The presence of extracellular DNA in the samples was investigated fluorometrically, using SYBR Green I as a stain, by the Thermo Scientific NanoDrop™ 3300 Fluorospectrometer (which allows to detect concentrations of DNA of 1-2 picograms in 1-2 µl of samples analyzed), and by qPCR analyses using the TaqMan technology targeting the 16S rDNA sequences. Both analyses revealed the lack of contamination of extracellular DNA (below detection limits) with intracellular DNA due to cell lysis.

We also carried out analyses on additional samples, collected in the continental margins of Mediterranean Sea (at 2348 m depth) and NE Atlantic Ocean (at 3668 m depth), to check the potential occurrence of cell lysis. Such analyses were based on the comparison between the amount of intracellular DNA actually extracted from the sediment samples, and the DNA amount estimated from the number of prokaryotic cells counted by epifluorescence microscopy before applying the protocol for the simultaneous extraction of extracellular and intracellular DNA1.

Since no significant differences were observed between the two DNA amounts, these findings reveal the lack of significant cell lysis (Figure S4).

All these findings allow us to exclude the release of extracellular DNA due to cell lysis induced by sample handling and storage and confirm the reliability of the protocol for the recovery of “genuine” extracellular DNA from marine sediments.

*Pyrosequencing analysis*

PCR reactions were performed in a volume of 50 µl in a thermalcycler (Biometra, Germany) using the MasterTaq® kit (Eppendorf AG, Germany), which reduces the effects of PCR-inhibiting contaminants. Thirty PCR-cycles were used, consisting of 94°C for 1 minute, 55°C for 1 minute and 72°C for 2 minute, preceded by 3 minutes of denaturation at 94°C and followed by a final extension of 10 minutes at 72°C. To check for eventual contamination of the PCR reagents, negative controls containing the PCR-reaction mixture but without the DNA template were run during each amplification. Positive controls, containing genomic DNA of *Escherichia coli* and *Methanocaldococcus jannaschii*, were also used. PCR-products were checked on agarose-TBE gel (1%), containing ethidium bromide for DNA staining and visualization.

Different PCR reactions were run for each sample and then combined together to reduce possible PCR biases and to reach the amount necessary for 454 analyses. The amplicons were purified using Amicon Ultra 50k device (Millipore). The amplicon length and concentration were estimated using the BioAnalyzer microfluidics device (Agilent), and then each amplicon was sequenced via emulsion PCR (performed using the recommended kit and protocol from 454 Life Sciences) by using a Genome Sequencer FLX Titanium (Roche).

**Supplementary tables**

**Table S1.** Environmental characteristics of sampling sites. Mean and standard deviations (±) are reported for protein, carbohydrate, lipid and biopolymeric C concentrations in the sediments investigated.

|  | *Mediterranean Sea* | *NE Atlantic Ocean 1* | *NE Atlantic Ocean 2* | *Arctic margin* |  |
| --- | --- | --- | --- | --- | --- |
| **Depth (m)** | 2342 | 3475 | 4902 | 2545 |  |
| **Latitude (N)** | 42.080 | 40.167 | 40.167 | 79.067 |  |
| **Longitude (E)** | 4.682 | 9.983 | 10.984 | 4.170 |  |
| **Sedimentation rate (cm y-1)** | 0.171 | 0.032 | 0.0033 | 0.0194 |  |
| **Temperature (°C)** | 13.10 | 2.57 | 2.47 | -0.84 |  |
| **Salinity** | 38.43 | 34.84 | 34.86 | 34.92 |  |
| **Proteins**  **(mg g-1)** | 1.88 ± 0.43 | 1.43 ± 0.17 | 1.41 ± 0.7 | 3.38 ± 0.59 |  |
| **Carbohydrates (mg g-1)** | 3.66 ± 0.79 | 1.20 ± 0.12 | 2.67 ± 0.25 | 4.96 ± 0.32 |  |
| **Lipids**  **(mg g-1)** | 0.70 ± 0.17 | 0.20 ± 0.05 | 0.33 ± 0.05 | 0.68 ± 0.15 |  |
| **Biopolymeric C (mg g-1)** | 2.4 ± 0.50 | 1.33 ± 0.17 | 2.01 ± 0.17 | 4.14 ± 0.53 |  |

1Buscail *et al*., 1997; 2 Garcia *et al.*, 2008; 3Carvalho *et al*., 2011; 4Carignan *et al.*, 2008

**Table S2.** Abundance of sequences for each sample considered in the present study at each step of quality check and during the QIIME analysis.

| *Pool* | *Sample* | *Raw sequences* | *After error correction and quality check* | *To the QIIME pipeline* | *Aligned* |
| --- | --- | --- | --- | --- | --- |
| **Extracellular DNA** | *Mediterranean Sea* | 6237 | 5159 | 4912 | 3440 |
|  | *NE Atlantic 1* | 7988 | 6599 | 6400 | 5469 |
|  | *NE Atlantic 2* | 13020 | 11488 | 8508 | 7671 |
|  | *Arctic margin* | 7857 | 6981 | 5877 | 4777 |
| **Intracellular DNA** | *NE Atlantic 1* | 10330 | 8410 | 8273 | 7533 |
|  | *NE Atlantic 2* | 10058 | 8469 | 8405 | 7268 |

**Table S3.** Comparison among extracellular DNA pools from different geographical areas after normalization to 3400 sequences. Reported are the OTU richness and the number of prokaryotic families identified within each sample.

| *Pool* | *Sample* | *OTU*  *Richness* | *Number of families* |
| --- | --- | --- | --- |
| **Extracellular DNA** | *Mediterranean Sea* | 1158 | 201 |
|  | *NE Atlantic 1* | 1157 | 236 |
|  | *NE Atlantic 2* | 1003 | 218 |
|  | *Arctic margin* | 1101 | 212 |

**Table S4.** Comparison between extracellular and intracellular DNA samples from the two sites of the NE Atlantic margin (sites 1 and 2) after normalization to 5000 sequences. Reported are the OTU richness and the number of prokaryotic families identified within each sample.

| *Pool* | *Sample* | *OTU*  *Richness* | *Number of families* |
| --- | --- | --- | --- |
| **Extracellular DNA** | *NE Atlantic 1* | 1398 | 228 |
|  | *NE Atlantic 2* | 1218 | 212 |
| **Intracellular DNA** | *NE Atlantic 1* | 1242 | 205 |
|  | *NE Atlantic 2* | 1207 | 207 |

**Supplementary figures**

**Figure S1.** Rarefaction curves after normalisation of: A) extracellular DNA samples from different geographical areas (Mediterranean, NE Atlantic and Arctic margins) and B) extracellular and intracellular DNA samples contextually extracted from NE Atlantic sites 1 and 2.

**
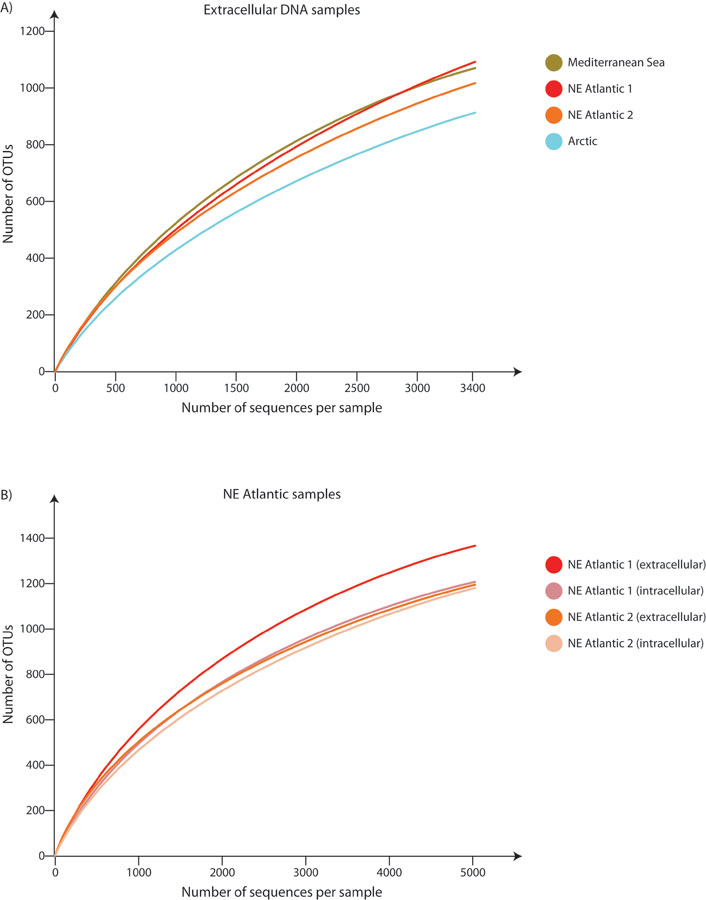
**

**Figure S2.** Output of the cluster analysis (based on the Jaccard dissimilarity index calculated on a presence/absence OTU matrix) carried out on the assemblage composition at OTU level of the extracellular DNA pools from the different continental margins investigated.


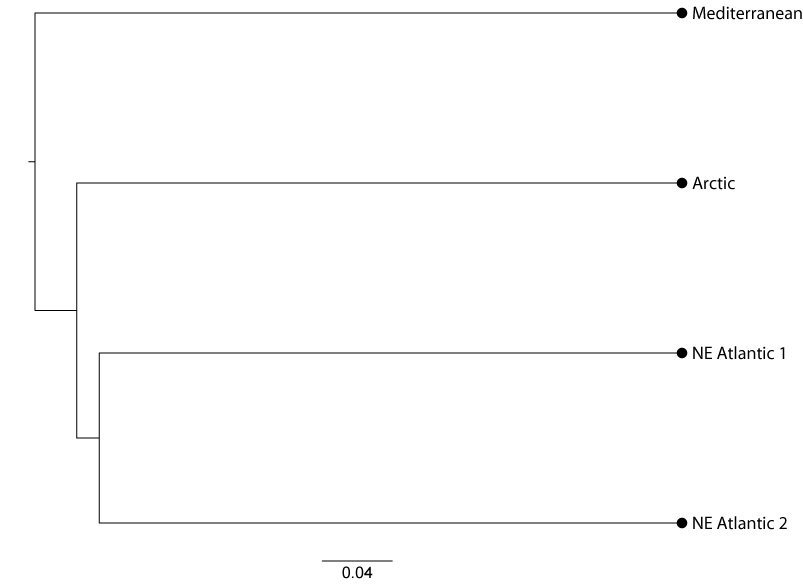


**Figure S3.** Prokaryotic assemblage structure determined in the intracellular DNA pools at the family level from the NE Atlantic margin. The taxa, whose OTUs contribute for at least 0.1% to the whole prokaryotic assemblages are shown.


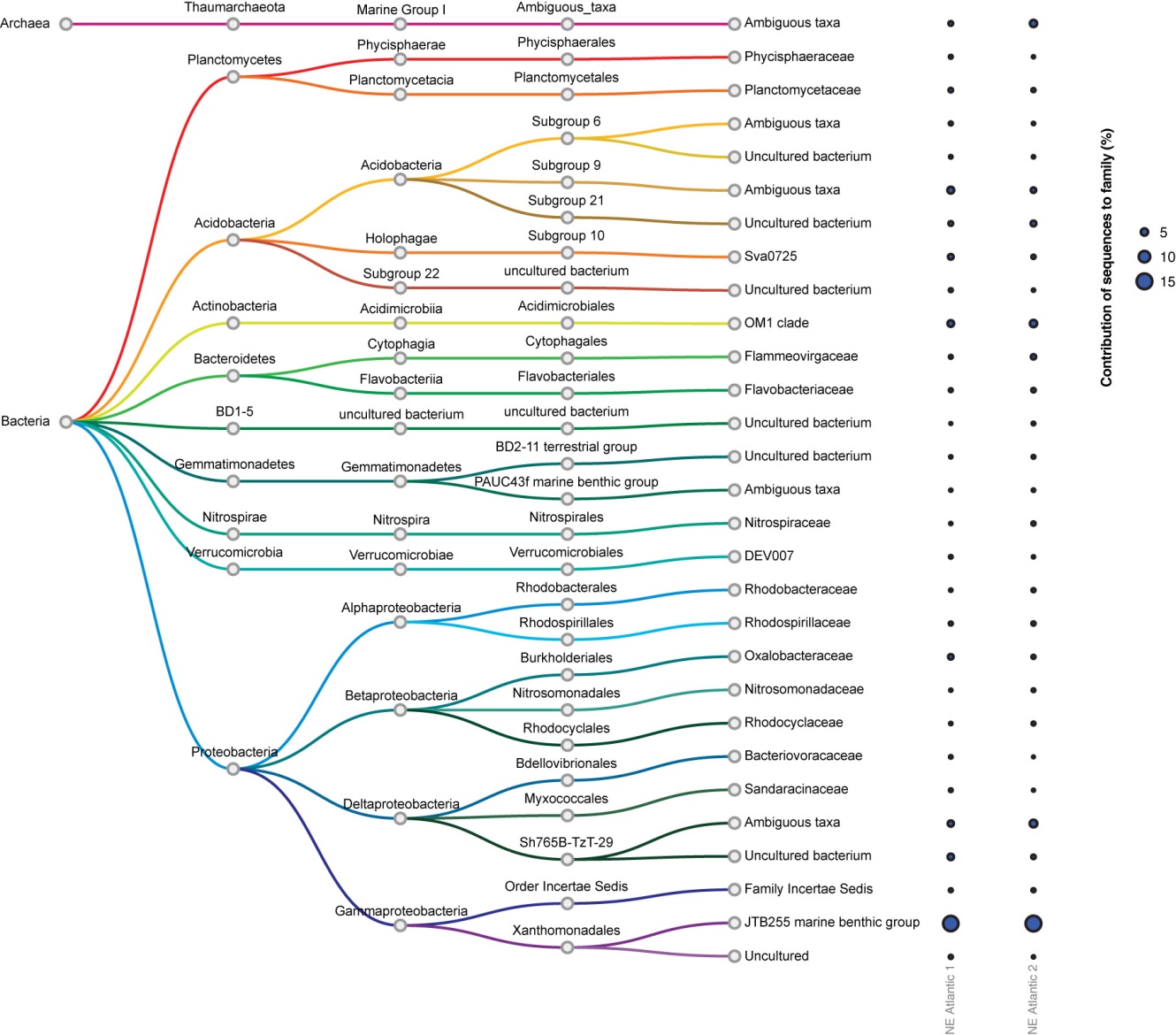


**Figure S4.** Comparison between the amount of intracellular DNA actually extracted from the sediment samples, and the DNA amount estimated from the number of prokaryotic cells counted by epifluorescence microscopy before applying the protocol for the simultaneous extraction of extracellular and intracellular DNA1.

**
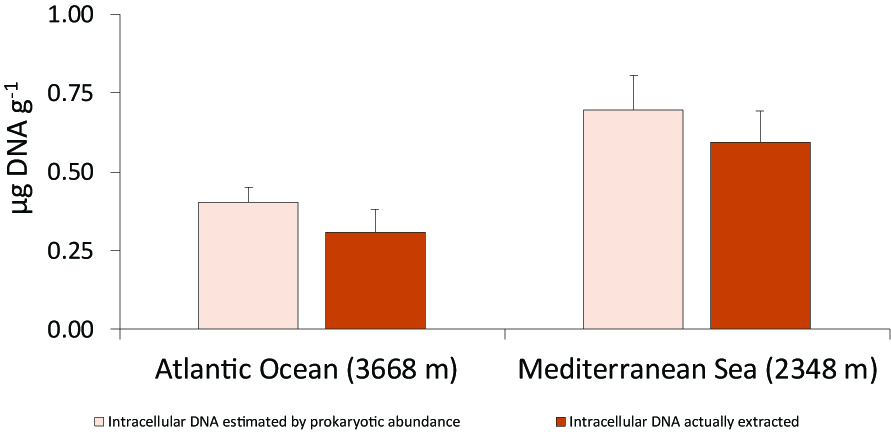
**

**Supplementary references**

c)

1. Corinaldesi, C., Danovaro, R. & Dell'Anno, A. Simultaneous recovery of extracellular and intracellular DNA suitable for molecular studies from marine sediments*. Appl Environ Microbiol.* **71**, 46-50 (2005).
2. Buscail, R., Ambatsian, P., Monaco, A. & Bernat, M. 210Pb, manganese and carbon: indicators of focusing processes on the northwestern Mediterranean continental margin. *Mar Geol*. **137**, 271-286 (1997).
3. Carignan, J., Hillaire-Marcel, C. & de Vernal, A. Arctic vs. North Atlantic water mass exchanges in Fram Strait from Pb isotopes in sediments. *Can J Earth Sci*. **45**, 1253-1263 (2008).
4. Carvalho, F. P., Oliveira, J. M. & Soares, A. M. 210Pb-excess and sediment accumulation rates at the Iberian continental margin. In: Isotopes in Hydrology, Marine Ecosystems and Climate Change Studies. 451-462 (2011).
5. Garcia, R. & Thomsen, L. Bioavailable organic matter in surface sediments of the Nazaré canyon and adjacent slope (Western Iberian Margin). *J Marine Syst*. **74**, 44-59 (2008).
